# The Role of Affectionate Caregiver Touch in Early Neurodevelopment and Parent–Infant Interactional Synchrony

**DOI:** 10.3389/fnins.2020.613378

**Published:** 2021-01-05

**Authors:** Sofia Carozza, Victoria Leong

**Affiliations:** ^1^Department of Physiology, Development and Neuroscience, Faculty of Biology, University of Cambridge, Cambridge, United Kingdom; ^2^Division of Psychology, Nanyang Technological University, Singapore, Singapore; ^3^Department of Psychology, University of Cambridge, Cambridge, United Kingdom

**Keywords:** touch, synchrony, social interaction, oxytocin, neurodevelopment, parent–infant

## Abstract

Though rarely included in studies of parent–infant interactions, affectionate touch plays a unique and vital role in infant development. Previous studies in human and rodent models have established that early and consistent affectionate touch from a caregiver confers wide-ranging and holistic benefits for infant psychosocial and neurophysiological development. We begin with an introduction to the neurophysiological pathways for the positive effects of touch. Then, we provide a brief review of how affectionate touch tunes the development of infant somatosensory, autonomic (stress regulation), and immune systems. Affective touch also plays a foundational role in the establishment of social affiliative bonds and early psychosocial behavior. These touch-related bonding effects are known to be mediated primarily by the oxytocin system, but touch also activates mesocorticolimbic dopamine and endogenous opioid systems which aid the development of social cognitive processes such as social learning and reward processing. We conclude by proposing a unique role for affectionate touch as an essential pathway to establishing and maintaining parent-infant interactional synchrony at behavioral and neural levels. The limitations of the current understanding of affectionate touch in infant development point to fruitful avenues for future research.

## Introduction

Early interactions with a parent provide the foundation for infant cognitive and socioemotional development (Raby et al., 2015; Mermelshtine and Barnes, 2016). Affectionate touch, which includes non-noxious light stroking, pressure, and holding, is a unique and essential feature of an infant’s interpersonal landscape (Hertenstein, 2002). The sensation of affectionate touch begins in the skin, where a variety of low-threshold mechanoreceptors (LTMRs) respond to different aspects of tactile stimulation. Some classes of LTMRs are myelinated, and their rapid transmission of sensory information enables the brain to discriminate physical stimuli as they touch the skin, while unmyelinated C afferents transmit information about slow and stable stimuli (McGlone et al., 2014; Olson et al., 2016). Among classes of cutaneous nerves, C-tactile afferents (CTs) appear to be particularly implicated in affectionate touch: light pressure and gentle stroking, especially at skin temperature, results in vigorous CT firing and is perceived as being most enjoyable or rewarding (Löken et al., 2009; Ackerley et al., 2014; Pawling et al., 2017). Importantly, children prefer CT-targeted touch throughout development, and when parents are asked to stroke their babies, they naturally do so at a velocity that optimally activates CTs (Croy et al., 2016, 2019). During face-to-face interactions, parent use of affectionate stroking decreases an infant’s arousal and increases positive emotionality (Peláez-Nogueras et al., 1997; Fairhurst et al., 2014). The impact of affectionate touch is far from transient; early interventions involving holding such as kangaroo care, or KMC, lead to positive physiological and psychological outcomes such as regulating stress reactivity and promoting mother–infant bonding, while the deprivation of touch is associated with a range of developmental deficits (Feldman et al., 2002a; Field, 2010b; Moore et al., 2016).

It is hypothesized that affectionate touch—both through CTs (Walker et al., 2017) and other nerve fibers (Uvnäs-Moberg et al., 2015)—achieves many of these effects through the release of the nonapeptide, oxytocin. Gentle stroking of rats increases Fos expression in the oxytocin-producing paraventricular nucleus of the hypothalamus (PVN), and increased plasma oxytocin levels are observed in rats following a wide variety of forms of peripheral sensory stimulation (Stock and Uvnäs-Moberg, 1988; Okabe et al., 2015). In humans, skin-to-skin contact (SSC) and KMC increase peripheral oxytocin levels in both parents and their infant (Vittner et al., 2019; Hardin et al., 2020). It is important to note that this effect is observed only when measured by enzyme immunoassay rather than radioimmunoassay, which may indicate a specific role for active fragments of oxytocin (Uvnäs Moberg et al., 2019); future studies should examine this discrepancy. As oxytocinergic projections from the PVN regulate regions throughout the brain involved in social interaction, pain, stress, and autonomic regulation (Uvnäs-Moberg et al., 2015), the oxytocinergic system is well-positioned to link touch to a wide range of physiological and psychosocial outcomes.

**FIGURE 1:**
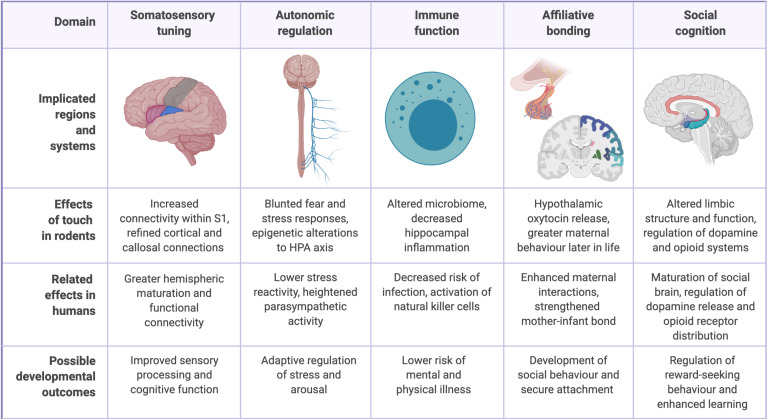
Physiological and psychological benefits of early-life caregiver touch.

While affectionate touch still remains conspicuously understudied, advances have been made in recent years through both human neuroimaging studies and animal models of maternal care, such as rodent licking and grooming (LG) behavior (Ardiel and Rankin, 2010; Botero et al., 2020). A complete understanding of the role of touch remains elusive, in part because of its dependence on the higher-level social and cultural dimensions of the human person (Ellingsen et al., 2016; Feldman, 2016). However, studies have linked affectionate caregiver touch to infant development across five interrelated domains: the somatosensory system, the autonomic system, immune function, affiliative bonding, and social cognition (Figure 1).

## Physiological Benefits of Affectionate Caregiver Touch

### Somatosensory Tuning

Affectionate caregiver touch in infancy may aid the maturation of the somatosensory system through experience-dependent plasticity. Most tactile information, including the location and intensity of pleasant touch, is processed in the somatosensory cortex (S1) while the valence and “pleasantness” of touch is detected by the posterior insular cortex (Lindgren et al., 2012; Gordon et al., 2013; Case et al., 2016; Morrison, 2016). The somatosensory processing pathway is highly organized, both serially between regions and somatotopically within regions (Ruben et al., 2001; Björnsdotter et al., 2009; Seelke et al., 2012). While its initial formation is based on genetic factors and sensory feedback from spontaneous movements (Milh et al., 2007; Antón-Bolaños et al., 2019), the system is subsequently refined through experience-dependent mechanisms of structural plasticity (Feldman and Brecht, 2005). In rodents, S1 matures soon after birth, and early-life contact is responsible for the refinement of both its intrinsic connectivity and its cortical and callosal projections (Seelke et al., 2016; Khazipov and Milh, 2018).

Affectionate touch may refine the somatosensory system in humans as well. The fundamental shift from spontaneous to evoked somatosensory activity occurs in the third trimester (Fabrizi et al., 2011), and after birth, the intensity and valence of touch experiences continue to shape somatosensory development (Maitre et al., 2017). In infants, gentle stroking preferentially activates both S1 and the posterior insular cortex; these regions are slightly different from those activated in adults, which may reflect an experience-dependent shift in the localization of affectionate touch processing (Jönsson et al., 2018; Pirazzoli et al., 2019; Tuulari et al., 2019). Lower-level processes of sensation and perception lay the foundation for development of higher-level cognitive functions, therefore early experiences of touch also contribute toward the maturation of neural circuitry for complex cognitive and social function. In fact, infants whose mothers use skin-to-skin touch show greater frontal alpha EEG asymmetry, a pattern of neural activity that reflects emotional processing and cognitive maturation (Hardin et al., 2020). Similarly, pre-term babies who receive caregiver skin contact show faster hemispheric maturation and improved functional connectivity (Scher et al., 2009; Schneider et al., 2012). In fact, an intervention of SSC paired with contingent vocalizations is currently under investigation for its effects on neonatal neurocognitive development (Neel et al., 2019). Conversely, institutionalized children—who often receive inconsistent and low-contact care—tend to exhibit sensory dysregulation, including heightened sensory reactivity, higher rates of sensory processing disorders, and some degree of touch aversion (Dozier et al., 2002; Wilbarger et al., 2010).

### Autonomic Regulation

Affectionate caregiver touch alters multiple aspects of autonomic function, including hypothalamic-pituitary-adrenal (HPA) axis activity and parasympathetic nervous tone. The HPA axis mediates the mammalian stress response. Rats raised with more parental LG show blunted responses to stress and attenuated fear of novelty, due to epigenetic alterations in the expression of hormone receptors in the hippocampus and amygdala (Liu et al., 1997; Caldji et al., 1998; Kundakovic and Champagne, 2015). These effects are specific to touch, as the experimental addition of gentle stroking in the absence of the dam results in the same changes in glucocorticoid receptor expression and corticosteroid secretion (Jutapakdeegul et al., 2003). Consistent with these findings, maternal touch—both during free play and KMC—decreases an infant’s physiological stress reactivity (Feldman et al., 2010; Hardin et al., 2020). There appears to be a sensitive period for this effect around the time of birth, as SSC between a mother and her neonate decreases salivary cortisol most when it takes place immediately after birth, and lower stress reactivity in infants is observed even a year later. This touch-mediated effect is hypothesized to be an innate way of reducing the infant’s “stress of being born” (Bystrova et al., 2007; Takahashi et al., 2011).

Touch likely achieves stress regulation through oxytocin release in the hypothalamus (Smith and Wang, 2014). Oxytocinergic projections from the PVN to regulatory regions in the brainstem, such as the nucleus of the solitary tract, reduce the activity of noradrenergic neurons and thereby upregulate parasympathetic nervous function (Uvnäs-Moberg et al., 2015). This is supported by the broad range of autonomic changes, beyond HPA activity, that are associated with touch. Throughout infancy, both maternal and paternal touch triggers cardiac deceleration, but only when it involves stroking at speeds that optimally activate CTs (Aguirre et al., 2019; Manzotti et al., 2019; Van Puyvelde et al., 2019). Similarly, neonatal SSC promotes heart rate stabilization and arousal regulation, as well as greater and more rapid weight gain (Feldman et al., 2002b; Cong et al., 2012; Samra et al., 2013). These effects are consistent with increased vagal tone, which modulates heart rate and the gut-brain axis (Yuan and Silberstein, 2016). In fact, there is evidence that stimulation of pressure receptors in the skin increases vagal activity, and that infant vagal activity increases during mother–child interactions (Field and Diego, 2008). Thus, an absence of caregiver touch could also lead to adverse mental and physical outcomes through decreased vagal tone (Thayer and Sternberg, 2006) (in parallel with the HPA effects described previously). In line with this explanation, the infants of depressed mothers—who use less affectionate touch—fail to show an increase in vagal tone over time and often exhibit developmental delays (Field et al., 1995; Herrera et al., 2004; Aoyagi and Tsuchiya, 2019; Mantis et al., 2019).

### Immune Function

A lack of early life-care, which includes touch deprivation, can lead to immune dysregulation. In rats, early isolation alters gut microbiota and raises levels of inflammatory molecules in the hippocampus, indicators of immune dysfunction that are linked to anxiety-like behaviors (Dunphy-Doherty et al., 2018). Children with a history of institutionalization experience a higher risk of intestinal and respiratory infections, as well as skin disorders (Rutter et al., 1998). While the deprivation of touch has yet to be studied in human immune development, childhood adversity is linked to elevated inflammation, T-cell proliferation, and impoverished cellular immune function (Fagundes et al., 2013), and greatly increases risk of physical and mental illness (Anda et al., 2006).

Several human studies link affectionate caregiver touch to positive immunological outcomes. The use of KMC decreases a neonate’s risk of severe infections, as well as a variety of other illnesses (Charpak et al., 2001; Boundy et al., 2016). The use of massage increases an infant’s proportion of active natural killer cells, although other immunological markers are unaffected (Ang et al., 2012). As microbiota are a critical component of the immune system that remains underdeveloped at birth, one possibility is that touch enhances infant immune function through the transferal of bacteria from the mother’s skin (Groer et al., 2015). However, given that massage leads to positive immunological outcomes in a range of populations (Field, 2010b), it is more likely that touch could act through a central neuroendocrine mechanism such as oxytocin release. Oxytocin itself plays a critical role in the regulation of the immune system, and has been implicated in the development of T-cells, the suppression of inflammatory cytokines, and wound healing, among other effects (Li et al., 2017; Uvnäs Moberg et al., 2019). While some immunological effects may be achieved through the activation of oxytocin receptors in peripheral organs, more directly, parvocellular neurons in the PVN and supraoptic nucleus have been shown to modulate inflammatory pain by releasing oxytocin in the nuclei of the brainstem and spinal cord (Eliava et al., 2016). As inflammatory cascades have been linked to the pathogenesis or prognosis of an increasing number of common chronic conditions (Bennett et al., 2018), oxytocinergic regulation of the immune system in general and inflammation in particular might provide one possible biological link between early-life touch and long-term health.

## Psychosocial Benefits of Affectionate Caregiver Touch

### Affiliative Bonding

Touch may contribute to the genesis of attachment between parent and infant through the action of oxytocin. In rats, gentle stroking increases Fos expression in the oxytocin-producing PVN, and maternal LG induces region-specific increases in oxytocin receptor expression (Francis et al., 2001; Okabe et al., 2015). While rodents do not form attachments as such, SSC with the dam—together with her olfactory cues—are signals of maternal care, and rats deprived of tactile and olfactory stimulation in infancy show impaired maternal behavior themselves in later life (Melo et al., 2006; Kojima et al., 2012). Both SSC and KMC appear to increase peripheral oxytocin levels in human infants, when measured with enzyme immunoassay (Vittner et al., 2018; Hardin et al., 2020). Repeated stimulation of oxytocin release in infants, and its attendant positive effects on autonomic function, may over time lead to a conditioned oxytocinergic response to maternal cues (Uvnäs-Moberg et al., 2015). Patterns of maternal engagement are also associated with different patterns of oxytocin receptor methylation in infants (Krol et al., 2019), which could modulate the development of infant social behavior (Gordon et al., 2011; Markova, 2018; Xu et al., 2019).

Moreover, affectionate touch is bidirectionally related to parent–infant bonding. In humans, mothers who perform KMC demonstrate heightened sensitivity toward their infant, as well as increased chances of a successful and long first breastfeeding (Tessier et al., 1998; Karimi et al., 2019). Breastfeeding, both through stimulation of sensory receptors in the skin and through the suckling stimulus, results in pulsatile oxytocin release in the mother that increases patterns of social interaction and attenuates pain and stress (UvnäsMoberg et al., 2020). Oxytocin increases parental responsiveness, as well as maternal milk production and consequent breastfeeding (Feldman and Eidelman, 2003; Vittner et al., 2018). In cases of maternal depression, mothers show difficulties in breastfeeding, blunted oxytocin release, and fewer face-to-face interactions with their infants, who are in turn at higher risk of insecure attachment (Lovejoy et al., 2000; Toth et al., 2009; Field, 2010a; Stuebe et al., 2013). As the mother–child bond is a strong antecedent of the child’s socio-emotional and cognitive development (Stams et al., 2002), contributions to affiliative bonding would be an important role for touch in infant development.

The parent–infant bond, in turn, appears to promote the effectiveness of affectionate touch. For instance, 9-month-old infants show greater parasympathetic responses to CT-targeted (stroking at 3 cm/s) in the presence of their parents (Aguirre et al., 2019). Furthermore, when 6–8-month-old infants are gently stroked, they prefer to watch their mother receive synchronous touch over asynchronous touch, but do not show such a preference when gazing at strangers (Maister et al., 2020). Thus, an infant’s experience of affectionate touch appears to be bidirectionally related to the parent–infant bond.

### Social Cognition: Social Learning and Reward Processing

A growing body of evidence links caregiver touch to social cognition and function. In young rodents and infant macaque monkeys, early handling and stroking is positively associated with later social learning (Lévy et al., 2003; van Hasselt et al., 2012; Simpson et al., 2019). In humans, children who receive more frequent maternal touch during play show a greater social orientation, or relative interest in faces compared with other objects (Reece et al., 2016). Finally, gentle caregiver stroking—but not other forms of touch—enables 4-month-old infants to recognize faces with an averted gaze, though they typically attend only to faces with a direct gaze (Della Longa et al., 2019). These changes may be mediated by the maturation of the social brain, as frequent maternal touch is associated with greater activity and connectivity of cortical regions implicated in social processing (Brauer et al., 2016).

Rats that experience less LG show suppressed synaptic plasticity and intrinsic excitability in the dorsal hippocampus, and heightened plasticity and excitability in the ventral hippocampus; these changes are related to anxiety-like behaviors (Nguyen et al., 2015). Conversely, early tactile stimulation in rodents triggers dendritic changes that increase the connectivity of the prefrontal cortex and amygdala, differences which are linked to heightened performance on learning tasks (Richards et al., 2012). Though comparable studies have not yet been conducted in humans, in general, parent–infant synchronous interactions have been identified as a key factor in the formation of reward circuitry (Feldman, 2017).

Early experiences of touch also regulate motivational salience through changes to the mesocorticolimbic dopamine and endogenous opioid systems. Within the central nervous system, dopamine release can be associated with both positive (e.g., motivational salience) and negative (e.g., stress response) effects (Trainor, 2011), but given the positive valence of its psychosocial context, affectionate caregiver touch likely activates the prior pathway. Maternal LG triggers a lasting increase in the number of dopaminergic neurons in the ventral tegmental area, particularly in nuclei connected to reward-related areas such as the ventral striatum and the amygdala (Peña et al., 2014). Furthermore, developmentally isolated rats show region-specific changes in dopamine turnover, while rats that receive more handling or grooming show heightened dopamine release in response to natural stimuli and blunted sensitivity to stress or psychostimulants (Heidbreder et al., 2000; Brake et al., 2004). Several studies in humans parallel these findings: pleasant touch triggers dopamine release, while less early-life care is associated with greater dopamine release in response to stress (Pruessner et al., 2004; Field et al., 2005). With regard to the opioid system, neonatal handling of rodents increases the expression of μ opioid receptors (MOR) in reward-related areas (Kiosterakis et al., 2009). Similarly, pleasant social touch increases the availability of MOR in human adults in the thalamus, striatum, and frontal, cingulate, and insular cortices (Nummenmaa et al., 2016). Therefore, affectionate touch appears to promote the development of neural circuits involved in social forms of learning, as well as motivation and reward processing.

## A Role for Touch in Parent–Infant Interactional Synchrony

Finally, affectionate touch may provide a unique and essential path to achieving synchrony during parent–infant interactions. Synchrony is the interpersonal coordination of behavioral and neurophysiological rhythms, a normative early-life experience that advances sensory processing, potentiates learning, regulates emotions and arousal, and promotes a stable attachment bond between the infant and her caregiver (Harrist and Waugh, 2002; Beebe et al., 2010; Wass et al., 2020). To facilitate synchrony, parents are primed to express a range of behaviors from the time of their infant’s birth, including infant directed speech, gaze, positive affect, and touch (Feldman, 2012). Most developmental research has focused on the vocal, visual, and affective modalities, because synchrony between humans can be established in the absence of physical contact (Feldman et al., 2011). However, SSC between parents and their neonate has been shown to increase the quantity and coordination of vocal and tactile interactions (Velandia et al., 2010). Here, we elaborate on two potential ways in which touch may contribute to achieving and maintaining parent–infant synchrony.

### Touch in Parent–Infant Communicative Rhythms

Touch may convey information that is necessary for the coordination of communicative rhythms between parent and infant. Previous research has found that mothers typically direct communicative behaviors toward their infant while the child is quiet but wakeful (Bell, 2020). As caregiver touch tends to provoke a state of calm alertness (Harrison et al., 2000), parents may use touch to ensure that their infants are available and ready to engage in patterns of communication. Continuous interpersonal touch may be more important during interactions with mothers, whose communicative patterns are usually more regular than the short arousing bursts typically used by fathers (Feldman, 2003). On the part of the infant, experiences of affectionate touch appear to support the development of bodily self-awareness, which in turn permits bodily attunement with their mothers (Montirosso and McGlone, 2020). Touch may perform this role by facilitating the coordination of sensory processing across modalities: in a recent study, infants detected and showed a preference for synchronous visuo-tactile stimuli over asynchronous stimuli while experiencing CT-targeted gentle stroking, but not during other touch (Della Longa et al., 2020). As interactional synchrony requires awareness of self and other, and takes place through coupled auditory, tactile, and visual signaling, affectionate touch may be a critical precursor to the infant’s detection of and participation in communicative rhythms.

### Touch in Parent–Infant Neural Synchrony

Touch may mediate the interpersonal coordination of neural activity. Specifically, parental social ostensive cues (typically gaze and speech, but also touch) are hypothesized to reset the phase of ongoing neural oscillations in an infant’s neocortex (Wass et al., 2020). This entrains the infant to the ongoing pattern of communication, and also aligns the oscillatory phases of parent and infant neural activity for optimal mutual receptivity. In this way, interpersonal social behaviors such as touch could trigger the synchronization of neural activity with concomitant benefits for communication and learning. A range of evidence supports this proposal along the visual and auditory modalities; both direct gaze and infant-directed speech generate unique patterns of cortical activity in infants (Zhang et al., 2011; Urakawa et al., 2015), and direct gaze produces temporally fine-grained neural entrainment between adults and infants (Leong et al., 2017). Maternal vocal and facial expression of positive emotion also increases parent–infant neural coupling more than negative affect (Santamaria et al., 2020). More broadly, mothers and their infants exhibit neural coupling during synchronous interactions, particularly in areas of the frontal cortex (Reindl et al., 2018; Nguyen et al., 2020). In adults, interpersonal touch such as hand holding enhances neural synchrony (Goldstein et al., 2018). While touch has yet to be isolated in studies of parent–infant neural synchrony, it appears probable that, like the ostensive cues of gaze and speech, touch leads to the phase reset and entrainment of neural oscillations.

## Conclusion and Future Directions

Experiences of affectionate touch, mediated by the CT system, are an essential component of parent–infant interactions. Affectionate touch appears to promote an infant’s somatosensory system development, autonomic regulation, parent–infant bonding and social development, reward processing and learning, and immune function. Future research should seek to clarify the neurobiological mechanisms involved in early experiences of affectionate touch, including the role of myelinated and unmyelinated sensory fibers and oxytocin signaling pathways in mediating the observed effects.

Affectionate touch may be particularly important for synchronous interactions, which involve the temporal coordination of behavior and physiology. Specifically, SSC, through afferent stimulation of nerves from the skin, may have a role in mutual synchronization of maternal and neonatal behaviors and neurophysiology, working in tandem with other important early social cues in the visual (e.g., eye contact) and auditory (e.g., infant-directed speech) sensory domains. Yet up until now, very little research on parent–infant synchrony has included assessments of touch, affectionate or otherwise. However, advances in naturalistic methods for parent–infant dyadic brain imaging (Georgieva et al., 2020; Noreika et al., 2020) now permit the fine-grained examination of complex social behavior involving multiple sensory modalities and their neural substrates (Neale et al., 2018). Therefore, the incorporation of affectionate touch as a key mechanism influencing bio-behavioral synchrony could provide a fruitful avenue for future research on parent–infant interactions.

Future research should also seek to clarify the relationship between affectionate touch and relevant parent and infant characteristics. More broadly, parent–infant interactions involving touch may vary based on the personality and temperament of the partners (Mammen et al., 2016; Beebe and Lachmann, 2017). Similarly, both touch and oxytocin alter brain activity in different ways depending on the relational context (Ellingsen et al., 2016; Baettig et al., 2019), which highlights the importance of including measures of attachment in studies of affectionate touch. Furthermore, a wide range of developmental disorders, including autism, have been bidirectionally linked to differences in touch interactions in infancy (Feldman et al., 2004; Cascio, 2010; Van keer et al., 2019; Provenzi et al., 2020). For example, in autism, atypical touch behavior is implicated as both a predictor of severity (e.g., children who show heightened tactile responsivity later develop greater autistic behaviors) and well as a potential compensatory mechanism (e.g., mothers of children with autism use more and longer-lasting physical proximity and touch to upregulate social engagement) (Doussard-Roosevelt et al., 2003; Saint-Georges et al., 2011; Mammen et al., 2015). These individual differences in early social sensory behavior could inform the development of sociometric markers for neuropsychiatric disorders in development (Leong and Schilbach, 2019). Finally, mothers touch their male and female infants differently, and a number of sex differences have been found in infant somatosensory processing, including in responsivity to SSC based on both the infant’s sex and that of the parent (Velandia et al., 2012; Fausto-Sterling et al., 2015; Walker et al., 2018). Therefore, measures of cognition, personality and temperament, attachment, developmental status, and sex should be incorporated in future studies of caregiver touch and its role in parent–infant interactional synchrony.

## Author Contributions

SC and VL wrote the manuscript. Both authors contributed to the article and approved the submitted version.

## Conflict of Interest

The authors declare that the research was conducted in the absence of any commercial or financial relationships that could be construed as a potential conflict of interest.

## Glossary of Abbreviations

CTs: C-tactile afferents. A class of unmyelinated sensory neurons found in human skin that mediate the sensation of pleasant touch.
HPA axis: the hypothalamic-pituitary-adrenal axis. A set of interactions between the hypothalamus, pituitary gland, and adrenal glands that controls the mammalian stress response.
KMC: Kangaroo Mother Care. A method of care for human infants that involves placing the child on the mother’s chest for access to breastfeeding and skin-to-skin contact.
LG: licking and grooming. Maternal care behaviors observed in rodents.
LTMRs: low-threshold mechanoreceptors. A class of somatosensory receptors that react to light touch.
PVN: the paraventricular nucleus of the hypothalamus. A region of the hypothalamus that regulates a range of homeostatic functions and contains a large population of oxytocin-secreting neurons.
SSC: skin-to-skin contact. A method of care for human infants that involves placing the naked infant on the mother’s bare chest soon after birth.
S1: the primary somatosensory cortex. An area of the cortex in the postcentral gyrus that receives inputs from the thalamus; it is the primary region responsible for processing somatic stimuli.
